# Albuminocytologic Dissociation and the Impact of Age-Adjusted Cerebrospinal Fluid Protein Levels in Guillain–Barré Syndrome

**DOI:** 10.3390/neurolint17020018

**Published:** 2025-01-24

**Authors:** Nithisha Thatikonda, Alexandru Lerint, Chaitra Takle, Xiang Fang, Chilvana Patel

**Affiliations:** 1 Department of Neurology, University of Texas Medical Branch, Galveston, TX 77550, USA; anlerint@utmb.edu (A.L.); sxfang@utmb.edu (X.F.); chipatel@utmb.edu (C.P.); 2 John Sealy School of Medicine, University of Texas Medical Branch, Galveston, TX 77550, USA; catakle@utmb.edu

**Keywords:** cerebrospinal fluid–total protein, Guillain–Barré Syndrome, age-adjusted upper limit values, albuminocytologic dissociation

## Abstract

**Background:** This study examined the impact of age-adjusted cerebrospinal fluid (CSF) protein levels on clinical characteristics, disease severity, and outcomes in Guillain–Barré Syndrome (GBS) patients. **Methods:** This retrospective study included 71 GBS patients at UTMB Galveston. Albuminocytologic dissociation (ACD) was defined as CSF–total protein (CSF-TP) >0.45 g/L with a cell count of <50 cells/L. Patients were grouped using the conventional cutoff (>0.45 g/L) and age-adjusted upper limits (URLs) for CSF-TP levels, comparing clinical, CSF, and electrophysiological characteristics across groups. **Results:** The mean age was 50 years (SD = 14.5). The mean age of patients with a CSF-TP > 45 g/L was higher (53 vs. 39 years, *p* = 0.000), whereas no such difference was noted using age-dependent URLs. Using age-adjusted CSF-TP URLs reduced the sensitivity for detecting ACD by 20%. CSF-TP > age-adjusted URLs were associated with lower MRC sum scores (39 vs. 47.43, *p* = 0.000), higher ICU admission rates (34% vs. 20%, *p* = 0.003), and the need for second-line treatment (41% vs. 17%, *p* = 0.049), and the trends were not observed with the conventional cutoff of 0.45 g/L. CSF-TP was an independent predictor of lower MRC sum scores (*p* = 0.009, 95% CI −0.058, −0.009) and higher GBS disability scores (*p* = 0.015, 95% CI 0.000, 0.004). **Conclusions:** ACD is a common finding in GBS, but normal protein levels do not exclude the diagnosis. Using age-adjusted URLs might improve specificity but reduce sensitivity for ACD detection, potentially increasing false negatives. CSF-TP levels exceeding age-adjusted URLs were more strongly associated with greater disease severity and poorer outcomes compared to the conventional cutoff of 0.45 g/L.

## 1. Introduction

Guillain–Barré Syndrome (GBS) is an acute immune-mediated disorder affecting the peripheral nervous system, particularly peripheral nerves and spinal roots. Diagnosis primarily relies on clinical evaluation, supported by neurophysiological testing and cerebrospinal fluid (CSF) analysis. The key laboratory markers for GBS are CSF protein (CSF-TP) levels and cell count. A hallmark finding is elevated CSF-TP levels with a normal white blood cell count, a phenomenon called albuminocytologic dissociation (ACD)*,* which reflects blood–nerve barrier disruption, increased intrathecal antibody production, or both [1,2,3]. ACD, first described in 1912 by Sicard and Foix, has historically been integral to diagnosing GBS [4]. Research indicates that CSF-TP levels are linked to the severity and prognosis of GBS and may vary across clinical variants and electrophysiological subtypes [5,6,7,8,9]. However, the clinical relevance of ACD in modern practice is less clear [10,11]. It is now understood that CSF-TP levels naturally increase with age [11,12,13,14,15]. Recent studies emphasize the importance of using age-adjusted upper reference limits (URLs) instead of the traditional protein cutoff of >0.45 g/L to define ACD [13,14,15,16,17]. One study reported that applying age-adjusted URLs reduced the number of inflammatory neuropathy cases classified as ACD by 35% [12]. In light of these evolving insights, our study investigates variations in CSF-TP levels and cell counts in GBS patients, examining how these metrics correlate with clinical characteristics, disease variants, electrophysiological subtypes, severity, and patient outcomes.

## 2. Methodology

### 2.1. Study Design

This retrospective observational study was conducted using data from 71 patients diagnosed with GBS at UTMB Galveston between 1 January 2015 and 1 June 2024. Approval was obtained from the institutional review board (IRB) of UTMB Galveston, and patient consent was waived by the IRB due to the retrospective nature of the study design.

### 2.2. Criteria for Inclusion and Exclusion

Patients were selected based on the following inclusion criteria: (1) all patients comprised men and women between the age groups of 18 and 80 diagnosed with GBS and admitted to the hospital; (2) all patients had complete CSF study results; (3) all patients met the Asbury et al. [1] diagnostic criteria, which include 1. progressive weakness of 2 or more limbs due to neuropathy, 2. areflexia, 3. disease course < 4 weeks, and 4. exclusion of other causes (vasculitis, PAN, SLE, Churg–Strauss syndrome, toxins, lead, botulism, diphtheria, porphyria, and cauda equine syndrome). Patients with incomplete CSF results and absent electrophysiological studies were excluded from the study.

### 2.3. Data Collection

Data on demographics, baseline clinical characteristics, comorbidities such as diabetes and spinal canal stenosis, antecedent triggers such as vaccination and infections, anti-ganglioside antibody status, CSF, and electrophysiological study findings were collected at study entry upon the chart review of all patients. The time to lumbar puncture (LP) was recorded in days. We classified patients into GBS clinical variants, including sensory, sensorimotor, motor, Miller Fisher syndrome (MFS), and MFS-GBS overlap, as defined by the International GBS Outcome Study (IGOS) [8]. Electrophysiological characteristics were evaluated using the Hadden classification, categorizing patients as demyelinating, axonal, equivocal, or inexcitable [18].

Data on CSF characteristics such as CSF-TP, glucose, and cell count were collected. The proportion of patients fulfilling the definition of ACD was assessed using different criteria sets, including the Brighton criteria (CSF-TP > 0.45 g/L and <50 cells/µL) [2], Asbury criteria (CSF-TP > 0.45 g/L and <10 cells/µL) [1], and common clinical practice (CSF-TP > 0.45 g/L and <5 cells/µL) [8]. The severity of GBS was determined by the Modified Rankin Scale (MRC) sum score upon admission, which is a routinely used scale in clinical practice for assessing muscle strength from Grade 5 (normal) to Grade 0 (no visible contraction) [19]. Muscle strength was assessed in six muscles in the upper and lower limbs on both sides, with scores ranging from 60 (normal) to 0 (quadriplegic).

Data on treatment details and disease course, such as the type of treatment received, whether treatment was initiated before LP, the need for second-line treatment, intensive care unit (ICU) admission, intubation requirement, progression to CIDP, and mortality rates, were collected. Disease outcomes were measured using the GBS disability score at the time of discharge, which is a widely accepted scoring system using the scale from 0 (a healthy state) to 6 (dead) [20].

### 2.4. Data Analysis

We used two types of reference limits to define the upper limit of normal CSF-TP levels: (1) conventional upper limit (URL) value of 45 g/L according to the Ausbury criteria [1] and (2) more recently published age-dependent URL values [11,12]. This URL, which adjusts for the patient’s age (in years), was determined in a large control group comprising more than 6000 CSF samples using the following equation [13].

CSF total protein lim = 0.124 + 0.0284 × age − 7.08 × 10^−4^ × age^2^ + 8.23 × 10^−6^ × age^3^ −3.35 × 10^−8^ × age^4^

Based on the above formula, the age-dependent URL values for CSF-TP used in our study are as follows: 18–30 years: 0.15–0.50 g/L; 30–50 years: 0.15–0.60 g/L; 50–80 years: 0.20–0.70 g/L; 80–200 years: 0.20–0.75 g/L [13,14,15,16].

Based on these two URL reference values, we divided patients into two different sets of groups each: one set with CSF-TP > 45 g/L vs. <45 g/L and another set with CSF-TP > age-dependent URL vs. <age-dependent URL values. We then examined the association between baseline clinical, CSF, and electrophysiological characteristics between these two groups individually and compared them to see if any differences exist between the two groups. For continuous variables, the Mann–Whitney U test was applied, while categorical variables were analyzed using the χ² test, with statistically significant results (*p* < 0.05). We finally applied regression analysis to find the predictive value of various CSF properties such as the CSF protein (g/L), CSF cell count, CSF glucose, and the presence of ACD on disease severity, treatment, and outcomes. The model was appropriately adjusted for age, sex, and the presence of comorbidities such as diabetes and spinal canal stenosis. Data analysis was performed using IBM SPSS Statistics, version 29.

### 2.5. Data Availability

The raw data and the original data of the main results of this study are avaibale as Supplementary Materials.

## 3. Results

### 3.1. Characteristics of the Cohort

A total of 71 GBS patients were included in the retrospective observational study. The baseline clinical and electrophysiological characteristics in relation to CSF-TP levels (both CSF-TP > 45 g/L and > age-dependent URL) were outlined in Table 1. The association between disease severity, treatment, and outcomes and CSF-TP levels (both protein > 45 g/L vs. >age-dependent URLs) were described in Table 2. The mean age of the patients was 50 years (SD = 14.5). The mean age of the patients with CSF-TP > 45 g/L was significantly higher compared to patients with CSF-TP < 45 g/L (53 vs. 39 years, *p* = 0.000), whereas no such difference was noted between the groups when age-dependent URL values of CSF-TP were used. In this study, 55% of the patients were females. The original data of the main results described in Table 1 are avialable through Supplementary Materials (S1).

In this study, 77% of patients had elevated CSF-TP (>45 g/L) and 75% of patients had ACD as per the Brighton criteria (Table 3). Based on the Asbury and Cornblath criteria, where cell counts of ≤10 cells/μL are considered compatible with typical GBS, only 68% fulfilled the definition of ACD. In addition, when we used a cutoff of <5 cells/μL, which is commonly used in clinical practice to define a normal CSF cell count, a lower percentage of people, namely, 59%, fulfilled the definition of ACD. When we applied age-dependent URL values, only 58% had elevated CSF-TP, and 55% had ACD as per the Brighton criteria. The proportion of patients who had ACD was reduced by 20% using age-dependent URL values for CSF-TP (Table 3).

### 3.2. Clinical and Electrophysiologic Characteristics in Relation to CSF-TP Levels

**Figure 1:**
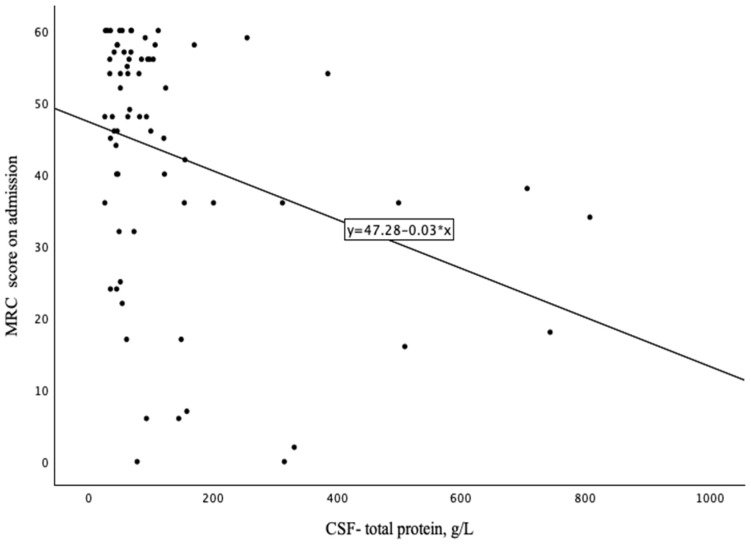
Scatter plot of CSF-TP levels and MRC sum score upon admission.

There was a moderate negative linear correlation found between CSF-TP levels and the MRC sum score upon admission (R^2^ = 0.34, p5% CI −0.53, −0.11, *p*= 0.004), meaning that the higher the CSF-TP, the lower the MRC sum score upon admission (Figure 1).

The mean MRC sum score on admission was 43.83. There are no differences observed in MRC sum scores between the groups of CSF-TP > 45 g/L and <45 g/L. However, patients with CSF-TP > age-dependent URLs had lower mean MRC scores compared to patients with CSF-TP < age-dependent URL values (39 vs. 47.43, *p* value = 0.000) (Table 2). The original data of the main results described in Table 1 are avialable through Supplementary Materials (S2).

Most patients, 56%, had a sensorimotor type of the clinical variant, followed by 13% with the sensory type, 11% with the pure motor type, 11% with the MFS-GBS overlap, and 9% with MFS. According to Hadden’s electrophysiological classification, 70% had a demyelinating subtype followed by 18% with the axonal type and 10% with the unequivocal type, and 1% had the inexcitable type, as observed in nerve conduction studies (Table 1).

### 3.3. Treatment and Outcomes in Relation to CSF-TP Levels

The majority of patients, 80%, were treated with IVIG as the first-line treatment. Of note, a considerable proportion of patients, 22 (31%), needed second-line treatment either with IVIG or PLEX. Out of these 22 (31%) patients who required second-line treatment, 17 had elevated CSF-TP > age-dependent URL values compared to only 5 patients with normal CSF-TP levels. This difference is statistically significant with *p* = 0.049. No such significant differences were found between the groups of elevated CSF-TP > 45 g/L and <45 g/L (Table 2).

In total, 20 (28%) patients required ICU admission, out of which 14 (34%) had elevated CSF-TP > age dependent URL values with a significant *p* value of 0.003. No such differences were noted between the groups of elevated CSF-TP > 45 g/L and <45 g/L (Table 2).

**Figure 2:**
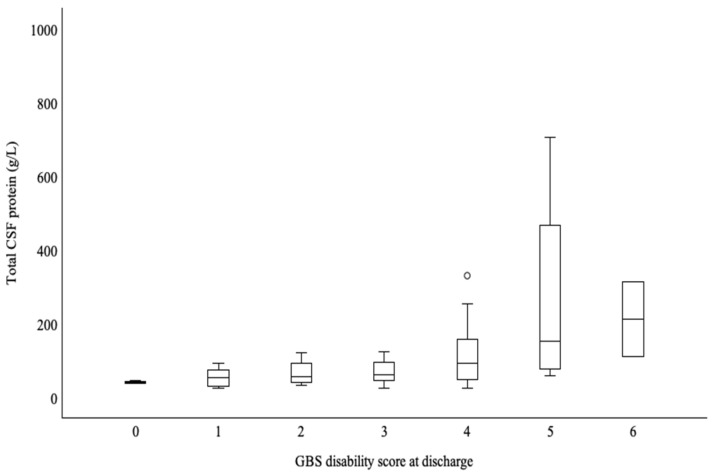
Variations in cerebrospinal fluid–total protein (CSF-TP) levels according to the GBS disability score.

Variations in CSF-TP levels according to GBS disability scores are shown in Figure 2. Patients with a high GBS disability score of ≥5 had higher CSF-TP levels (mean 265 g/L, SD = 216) when compared to patients with scores of <5 (mean 115 g/L, SD 135) (Figure 2).

The mean value of the GBS disability score at discharge was 3.32 (Table 1). Patients with elevated CSF-TP > 45 g/L had higher mean GBS disability scores of 3.54 compared to the CSF-TP < 45 g/L group with a *p* value of 0.0266. This *p* value becomes much smaller at 0.001 when differences in the mean GBS disability scores were analyzed with CSF-TP age-dependent URL values (3.75 in CSF-TP > age-dependent URL values group vs. 2.73 in CSF-TP < age-dependent URL values) (Table 2).

We applied multiple logistic regression to find the predictive value of various CSF properties on GBS disease severity, treatment, and outcomes (Table 4). The model was adjusted for factors such as age, sex, and a history of diabetes. CSF-TP levels were an independent predictor of low MRC sum scores on admission (*p* = 0.009, odds ratio −0.328, 95% CI −0.058, −0.009) and higher GBS disability scores (*p* = 0.015, odds ratio 0.304, 95% CI 0.000, 0.004) (Table 3). Other SCF properties, such as cell count, glucose, and the presence of ACD, were unable to predict GBS disease severity, treatment, and outcomes. The original data of the main results described in Table 1 are avialable through Supplementary Materials (S3).

## 4. Discussion

Our study investigated variations in CSF characteristics and their correlations with clinical characteristics, disease variants, electrophysiological subtypes, severity, and outcomes in GBS patients.

The mean age of the patients was 50 years (SD = 14.5). Patients with CSF-TP levels > 45 g/L had a significantly higher mean age compared to those with CSF-TP levels < 45 g/L (53 vs. 39 years, *p* = 0.000) (Table 1). However, when age-dependent upper reference limits (URLs) for CSF-TP were applied, this difference was no longer observed, indicating that CSF-TP levels are influenced by patient age. This finding aligns with recent observational studies conducted on a large cohort of Guillain–Barré syndrome (GBS) patients in Canada, which analyzed over 20,000 CSF samples over two decades [13]. These studies proposed new age-adjusted URLs for CSF-TP that were presumably more accurate than the conventional cutoff of 0.45 g/L. The revised age-adjusted URLs for CSF-TP have since been adopted in numerous studies [8,13,14,15,16,17].

Using the conventional cutoff of 0.45 g/L, 75% of patients were classified as having ACD. In contrast, only 55% were classified as having ACD when age-adjusted URLs for CSF-TP were applied (Table 3). The use of age-adjusted URLs led to a 20% reduction in the proportion of patients classified with ACD. Similar findings were reported in previous studies, where applying age-adjusted URLs reduced inflammatory neuropathy cases classified as ACD by 35% [12] and GBS cases classified as ACD by 21% [8]. The primary aim of adopting age-adjusted URLs is to improve specificity by reducing the over-diagnosis of elevated CSF-TP levels [11,12]. However, in our study, all patients had a confirmed diagnosis of GBS based on electrophysiological criteria. Applying age-adjusted URLs, therefore, resulted in a 20% reduction in the sensitivity for detecting ACD.

More than half of the patients, 56%, had a sensorimotor variant of GBS, and 70% of the patients had the demyelinating type of GBS according to the Hadden electrophysiological criteria. This is in accordance with the existing literature stating that these two are the most common types [8,9,21].

Though using age-adjusted URLs for CSF-TP revealed reduced sensitivity, it highlighted significant differences between patient groups when compared to the conventional cutoff value of 0.45 g/L. Notably, no differences were observed in the MRC sum scores between patients classified by the conventional cutoff of CSF-TP > 0.45 g/L vs. <0.45 g/L (46 vs. 41; *p* = 0.6). However, patients with CSF-TP levels > age-adjusted URLs had significantly lower mean MRC scores compared to those below the age-adjusted thresholds (39 vs. 47.43; *p* < 0.001). A similar trend was evident in ICU admission rates and the need for second-line treatments. Among the 20 patients (28%) who required ICU admission, 14 (34%) had elevated CSF-TP levels above age-adjusted URLs, with a statistically significant *p*-value of 0.003. Furthermore, of the 22 patients (31%) who required second-line treatment, 17 (24%) had CSF-TP levels exceeding age-adjusted URLs (*p* = 0.049).

Overall, CSF-TP > age-adjusted URLs were associated with lower MRC sum scores, higher ICU admission rates, and increased need for second-line treatments. These differences were not observed when using the conventional cutoff value of 0.45 g/L (Table 2). Notably, these findings could not be compared to prior studies, as no previous research has specifically examined the impact of age-adjusted CSF-TP levels in patients with GBS.

CSF-TP levels exceeding 45 g/L were associated with higher mean GBS disability scores (3.54 vs. 2.56; *p* = 0.026). This association was even more pronounced with age-adjusted URLs, showing a higher mean score and a more significant *p*-value (3.75 vs. 2.73; *p* = 0.001). As with earlier findings, these results could not be compared to previous studies, as no prior research has specifically investigated the effect of age-adjusted CSF-TP levels in GBS patients.

The following observations were noted when interpreting CSF-TP as a continuous variable. The mean MRC sum score on admission was 43.83. A moderate negative linear correlation was found between CSF-TP levels and the MRC sum score on admission (R^2^ = 0.34, p5% CI −0.53, −0.11, *p* = 0.004), meaning that the higher the CSF-TP, the lower the MRC sum score on admission (Figure 1). This finding is in accordance with a recently published study [8,9].

Finally, we applied multiple logistic regression with CSF-TP as a continuous variable (Table 4). The CSF-TP level was an independent predictor of low MRC sum scores on admission (*p* 0.009, odds ratio −0.328, 95% CI −0.058, −0.009) and higher GBS disability scores (*p* 0.015, odds ratio 0.304, 95% CI 0.000, 0.004). However, these findings cannot be compared to prior research due to the paucity of similar studies. Other CSF properties such as cell count, glucose, and the presence of ACD were unable to predict GBS disease severity, treatment, and outcomes.

In summary, our study confirmed that CSF-TP levels are influenced by patient age. Using age-adjusted CSF-TP URLs reduced the sensitivity for detecting ACD by 20%. CSF-TP levels > age-adjusted URLs were associated with lower MRC sum scores, higher ICU admission rates, and the need for second-line treatment, trends not observed with the conventional cutoff of 0.45 g/L. Additionally, CSF-TP > 45 g/L correlated with higher mean GBS disability scores, an association that was even stronger with age-adjusted URLs. Importantly, CSF-TP is an independent predictor of lower MRC sum scores on admission and higher GBS disability scores. In contrast, other CSF properties, including cell count, glucose levels, and the presence of ACD, were not predictive of GBS disease severity, treatment requirements, or outcomes.

Our study is the first, to our knowledge, to examine the correlation between CSF-TP levels and GBS disease severity and outcomes using age-adjusted URLs for CSF-TP. It is also the first to compare patient groups classified by age-adjusted URLs and the conventional cutoff of 0.45 g/L for CSF-TP.

However, our study has several limitations. It was a single-center, retrospective study with a small sample size of 71 patients. Although no significant differences were observed between groups based on the timing of lumbar puncture (LP) from symptom onset, the potential influence of this factor on CSF-TP levels cannot be fully excluded. Furthermore, a substantial proportion of patients lacked spinal imaging, leaving the contribution of spinal canal stenosis to elevated CSF-TP levels unresolved. Lastly, as age-adjusted URLs for CSF-TP are newly proposed, there are no prior studies available for direct comparison. Our findings require validation through future studies with larger sample sizes and prospective designs.

## 5. Conclusions

Patient age should be factored in when interpreting elevated CSF-TP values in GBS patients. While using age-adjusted URLs for CSF-TP offers a predictable improvement in specificity, the associated reduction in sensitivity for detecting ACD must be carefully weighed in clinical practice to minimize false-negative diagnoses. CSF-TP levels exceeding age-adjusted URLs were more strongly associated with greater disease severity and poorer outcomes compared to the conventional cutoff of 0.45 g/L. Additionally, absolute CSF-TP values should be considered when evaluating clinical outcomes in GBS patients, rather than solely focusing on the presence or absence of ACD.

## Figures and Tables

**Table 1:** Association between baseline clinical and electrophysiological characteristics and CSF-TP levels (both protein > 45 g/L vs. >age-dependent URLs).

Clinical Characteristics	Total Cohort	CSF-TP ≤0.45 g/L	CSF-TP >0.45 g/L	*p* Value	CSF-TP ≤ Age-Dependent URL	CSF-TP > Age-Dependent URL *	*p* Value
N	71	16 (23%)	55 (77%)		30 (42%)	41 (58%)	
Age, y	49.88	39.375	52.94	**0.000**	47.16	51.87	0.109
Sex				0.684			0.052
Male	32 (45%)	6 (37%)	26 (47%)		9 (30%)	23 (56%)	
Female	39 (55%)	10 (63%)	29 (53%)		21 (70%)	18 (44%)	
Ethnicity				0.378			0.816
Caucasian	41 (58%)	7 (44%)	34(62%		18 (60%)	23 (56%)	
African American	12 (17%)	3 (19%)	9 (16%)		5 (17%)	7 (17%)	
Hispanic	18 (25%)	6 (37.5%)	12 (22%)		7 (23%)	11 (27%)	
Antecedent triggers				0.664			0.491
URTI	16 (23%)	4 (25%)	12 (22%)		8 (27%)	8 (20%)	
GI/GU infection	17 (24%)	5 (31%)	12 (22%)		9 (30%)	8 (20%)	
Post-vaccination	2 (3%)	0 (0%)	2 (4%)		1 (3%)	1 (2%)	
Unknown	36 (51%)	7 (44%)	29 (53%)		12 (40%)	24 (59%)	
Diabetes	15 (21%)	1 (6%)	14 (25%)	0.191	2 (7%)	13 (32%)	**0.024**
Spinal canal stenosis	n = 49			0.119			0.313
No	23 (32%)	11 (69%)	12 (22%)		11 (37%)	12 (29%)	
Mild to moderate	25 (35%)	10 (63%)	15 (27%)		10 (33%)	15 (37%)	
Severe	1 (1%)	0 (0%)	1 (2%)		0 (0%)	1 (2%)	
Clinical variant types				0.086			**0.008**
Sensory	9 (13%)	3 (19%)	6 (11%)		6 (20%)	3 (7%)	
Sensorimotor	40 (56%)	9 (56%)	31 (56%)		15 (50%)	25 (61%)	
Motor	8 (11%)	4 (25%)	4 (7%)		7 (23%)	1 (2%)	
MFS	6 (8%)	0 (0%)	6 (11%)		1 (3%)	5 (12%)	
MFS-GBS	8 (11%)	0 (0%)	8 (15%)		1 (3%)	7 (17%)	
Hadden classification				0.890			0.084
Demyelinating	50 (70%)	12 (75%)	38 (69%)		17 (57%)	33 (80%)	
Axonal	13 (18%)	3 (19%)	10 (18%)		9 (30%)	4 (10%)	
Unequivocal	7 (10%)	1 (6%)	6 (11%)		4 (13%)	3 (7%)	
In excitable	1 (1%)	0 (0%)	1 (2%)		0 (0%)	1 (2%)	
Positive ganglioside antibody/s	18 (25%)	4 (25%)	14 (25%)	0.079	5 (17%)	13 (32%)	0.245
Time to LP, days	14.02	9.66	15.24	0.294	13.58	14.35	0.742

* Age-dependent upper limit (URL) reference values: 18–30 years: 0.15–0.50 g/L; 30–50 years: 0.15–0.60 g/L; 50–80 years: 0.20–0.70 g/L; 80–200 years: 0.20–0.75 g/L. For continuous variables, the Mann–Whitney U test was applied to test the association between clinical and neurophysiological characteristics and ACD. For categorical variables, the χ^2^ test was applied. Statistically significant results with *p* < 0.05 are marked in bold. Abbreviations: CSF, cerebrospinal fluid; MFS, Miller Fisher syndrome; GBS, Guillain–Barre syndrome; LP, lumbar puncture.

**Table 2:** Association between disease severity, treatment, and outcomes and CSF-TP levels (both protein > 45 g/L vs. > age-dependent URLs).

Clinical Characteristics	Total Cohort	CSF-TP ≤0.45 g/L	CSF-TP >0.45 g/L	*p* Value	CSF-TP ≤ Age-Dependent URL	CSF-TP > Age-Dependent URL *	*p* Value
MRC sum score on admission	43.83	46.13	41.53	0.600	47.43	39	**<0.000**
Treatment				0.221			0.858
IVIG	57 (80%)	13 (81%)	44 (80%)		24 (80%)	32 (78%)	
PLEX	14 (20%)	3 (19%)	11 (20%)		4 (13%)	10 (24%)	
Treatment initiated before LP	23 (32%)	5 (31%)	18 (33%)	0.996	13 (43%)	20 (49%)	0.831
Needed 2^nd^-line treatment	22 (31%)	1 (6%)	21 (38%)	0.033	5 (17%)	17 (41%)	**0.049**
ICU admission	20 (28%)	3 (19%)	17 (31%)	0.066	6 (20%)	14 (34%)	**0.003**
Required intubation	19 (27%)	1 (6%)	18 (33%)	0.074	4 (13%)	15 (37%)	0.056
Mortality	4 (6%)	0 (0%)	4 (7%)	0.621	0 (0%)	4 (10%)	0.215
GBS disability score at discharge	3.32	2.56	3.54	**0.026**	2.73	3.75	**0.001**
Progressed to CIDP	16 (23%)	2 (13%)	14 (25%)	0.581	6 (20%)	10 (24%)	0.881

* Age-dependent upper limit (URL) reference values: 18–30 years: 0.15–0.50 g/L; 30–50 years: 0.15–0.60 g/L; 50–80 years: 0.20–0.70 g/L; 80–200 years: 0.20–0.75 g/L. For continuous variables, the Mann–Whitney U test was applied to test the association between clinical and neurophysiological characteristics and ACD. For categorical variables, the χ^2^ test was applied. Statistically significant results with *p* < 0.05 are marked in bold. Abbreviations: CSF, cerebrospinal fluid; GBS, Guillain–Barre syndrome; IVIG, intravenous immunoglobulin; PLEX, plasmapheresis; CIDP, chronic inflammatory demyelinating polyneuropathy, LP, lumbar puncture; MRC, medical research council.

**Table 3:** Proportion fulfilling the definition of albuminocytologic dissociation (ACD) based on different criteria sets.

CSF-TP	All	<50 (Brighton Criteria)	<10 (Asbury Criteria)	<5 ^a^ (Common Clinical Practice)
>45 g/L	55 (77%)	53 (75%)	48 (68%)	42 (59%)
>age-dependent URL values *	41 (58%)	39 (55%)	35 (49%)	28 (39%)

Data are n (%). Abbreviation: CSF-TP = CSF total protein. ^a^ Commonly used in clinical practice.* Age-dependent upper limit (URL) reference values: 18–30 years: 0.15–0.50 g/L; 30–50 years: 0.15–0.60 g/L; 50–80 years: 0.20–0.70 g/L; 80–200 years: 0.20–0.75 g/L.

**Table 4:** Regression analysis showing the predictive ability of various CSF properties on GBS disease severity, treatment, and outcomes.

	Coefficient	SE	*p* Value	Odds Ratio	95% CI
MRC score on admission					
CSF protein, g/L	−0.033	9.632	**0.009**	−0.328	−0.058, −0.009
CSF cell count	0.013	0.043	0.765	0.039	−0.073, 0.099
CSF glucose	−0.076	0.093	0.415	−0.111	−0.262, 0.109
ACD, Brighton criteria	1.895	5.287	0.721	0.048	−8.671, 12.461
Constant	50.225	9.632	<0.001	30.977	69.473
ICU admission					
CSF protein, g/L	0.003	0.002	0.093	1.003	0.999, 1.007
CSF cell count	0.027	0.032	0.394	1.028	0.965, 1.095
CSF glucose	0.018	0.013	0.166	1.018	0.992, 1.045
ACD, Brighton criteria	1.053	0.798	0.187	2.867	0.600, 13.705
Constant	−2.889	1.693	0.088	0.056	
Required intubation					
CSF protein, g/L	0.004	0.002	0.059	1.004	1.000, 1.007
CSF cell count	0.040	0.036	0.265	1.040	0.970, 1.115
CSF glucose	0.016	0.014	0.253	1.016	0.989, 1.043
ACD, Brighton criteria	1.291	1.153	0.263	3.637	0.380, 34.836
Constant	−3.855	2.039	0.059	0.021	
Needed 2nd-line treatment					
CSF protein, g/L	0.002	0.002	0.228	1.002	0.999, 1.006
CSF cell count	0.039	0.039	0.312	1.040	0.964, 1.123
CSF glucose	0.016	0.014	0.274	1.016	0.988, 1.045
ACD, Brighton criteria	1.844	1.134	0.104	5.191	0.684, 58.398
Constant	−5.229	2.151	0.015	0.005	
GBS disability score at discharge					
CSF protein, g/L	0.002	0.001	**0.015**	0.304	0.000, 0.004
CSF cell count	0.005	0.003	0.185	0.176	−0.002, 0.012
CSF glucose	−0.008	0.007	0.259	−0.155	−0.023, 0.006
ACD, Brighton criteria	0.801	0.422	0.062	0.256	−0.043, 1.644
Constant	2.872	0.769	<0.001		1.335, 4.409
Mortality					
CSF protein, g/L	0.009	0.006	0.119	1.009	0.998, 1.020
CSF cell count	0.015	0.046	0.737	1.016	0.928, 1.111
CSF glucose	0.047	0.041	0.247	1.048	0.968, 1.135
ACD, Brighton criteria	2.930	13.993	0.834	18.720	0.000, 1.525 × 10^13^
Constant	−3.674	15.231	0.274	0.000	
Progressed to CIDP					
CSF protein, g/L	0.001	0.002	0.773	1.001	0.997, 1.004
CSF cell count	0.005	0.012	0.671	1.005	0.981, 1.030
CSF glucose	−0.460	0.025	0.066	0.955	0.909, 1.003
ACD, Brighton criteria	1.338	0.937	0.153	3.813	0.608, 23.917
Constant	0.126	2.173	0.954	1.134	

For continuous dependent variables, multiple linear regression test was applied. For categorical dependent variables, binary logistic regression was applied. Model was adjusted for age, sex, and diabetes. Statistically significant results with *p* < 0.05 are marked bold. Abbreviations: CSF, cerebrospinal fluid; GBS, Guillain–Barre syndrome; CIDP, chronic inflammatory demyelinating polyneuropathy; MRC, medical research council.

## Data Availability

The raw data and the original data of the main results of this study are available as Supplementary Materials.

## Supplementary Materials

The following supporting information can be downloaded at: https://www.mdpi.com/article/10.3390/neurolint17020018/s1; Table S1: original data of main results decribed in Table S1; Table S2: original data of main results described in Table S2; Table S3: original data main results described in Table S3.
