# Supplementary material for: Albuminocytologic Dissociation and the Impact of Age-Adjusted Cerebrospinal Fluid Protein Levels in Guillain–Barré Syndrome
Source: Neurol Int. 2025 Jan 24;17(2):18. doi: 10.3390/neurolint17020018 (PMC11858027; doi:10.3390/neurolint17020018)
Supplement: Supplementary file 1 [file neurolint-17-00018-s001.zip › neurolint-3380001-supplementary.pdf]

Table S1. Original data of the main results described in Table S1.

Clinical variant types and CSF-TP protein > age-dependent URL).

Chi-Square Tests			
	**Value**	**df**	Asymptotic Significance (2-sided)
Pearson Chi-Square	13.794[a]	4	0.008
Likelihood Ratio	14.870	4	0.005
Linear-by-Linear Association	3.260	1	0.071
N of Valid Cases	71		

a. 7 cells (70.0%) have expected count less than 5. The minimum expected count is 2.54.

Symmetric Measures			
		**Value**	**Approximate Significance**
Nominal by Nominal	Phi	.441	.008
	Cramer's V	.441	.008
N of Valid Cases		71	

Mann-Whitney U tests for signficant results described in in Table S1

Age and CSF-TP protein > 45 g/L.

Independent-Samples Mann-Whitney U Test Summary	
**Total N**	71
Mann-Whitney U	687.500
Wilcoxon W	2227.500
Test Statistic	687.500
Standard Error	72.634
Standardized Test Statistic	3.407
Asymptotic Sig.(2-sided test)	<.001

 https://doi.org/10.3390/neurolint17020018

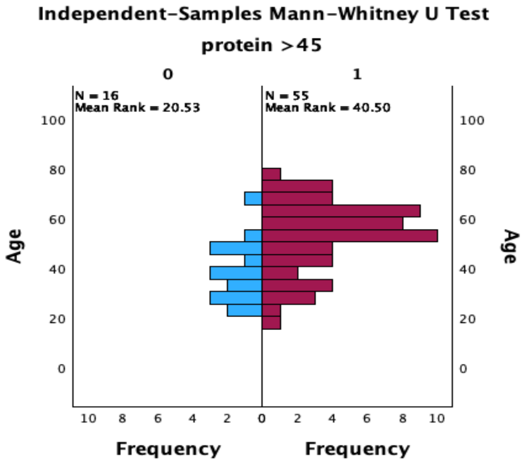
Independent-Samples Mann-Whitney U Test
protein >45
0
1
N = 16
Mean Rank = 20.53
N = 55
Mean Rank = 40.50
Age
Age
100
80
60
40
20
0
10 8 6 4 2 0 2 4 6 8 10
Frequency
Frequency

Age and CSF-TP > age-dependent URL.

Independent-Samples Mann-Whitney U Test Summary	
**Total N**	69
Mann-Whitney U	477.500
Wilcoxon W	1962.500
Test Statistic	477.500
Standard Error	68.619
Standardized Test Statistic	1.057
Asymptotic Sig.(2-sided test)	0.291

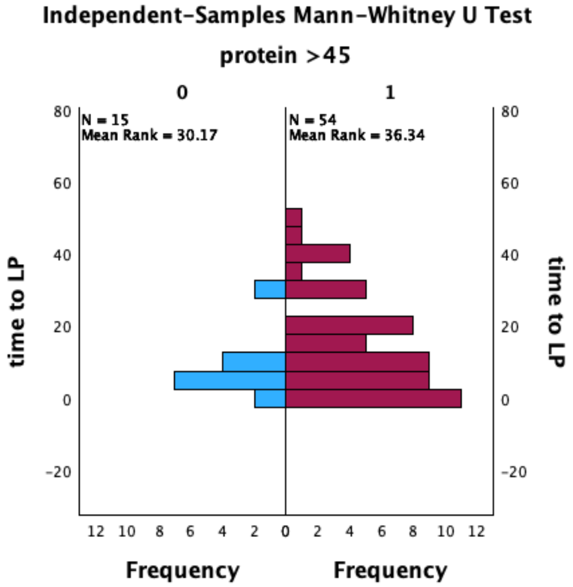

Table S2. Original data of the main results described in Table S2.

Chi-square tests for signficant results described in Table S2.

Needed 2nd line t/t and CSF-TP protein > 45 g/L.

Chi-Square Tests					
	**Value**	**df**	**Asymptotic Significance (2-sided)**	**Exact Sig. (2-sided)**	Exact Sig. (1-sided)
Pearson Chi-Square	5.910[a]	1	0.015		
Continuity Correction[b]	4.511	1	0.033		
Likelihood Ratio	7.271	1	0.007		
Fisher's Exact Test				0.015	0.012
Linear-by-Linear Association	5.827	1	0.016		
N of Valid Cases	71				

a. 1 cells (25.0%) have expected count less than 5. The minimum expected count is 4.96.

b. Computed only for a 2x2 table.

Symmetric Measures			
		**Value**	**Approximate Significance**
Nominal by Nominal	Phi	0.289	0.015
	Cramer's V	0.289	0.015
N of Valid Cases		71	

Needed 2nd line t/t and CSf-TP > age-dependent URL.

Chi-Square Tests					
	**Value**	**df**	**Asymptotic Significance (2-sided)**	**Exact Sig. (2-sided)**	Exact Sig. (1-sided)
Pearson Chi-Square	4.981[a]	1	0.026		
Continuity Correction[b]	3.889	1	0.049		
Likelihood Ratio	5.226	1	0.022		
Fisher's Exact Test				0.037	0.023
Linear-by-Linear Association	4.911	1	0.027		
N of Valid Cases	71				

a. 0 cells (0.0%) have expected count less than 5. The minimum expected count is 9.30. b. Computed only for a 2x2 table.

Symmetric Measures			
		**Value**	**Approximate Significance**
Nominal by Nominal	Phi	0.265	0.026
	Cramer's V	0.265	0.026
N of Valid Cases		71	

ICU admission and CSF-TP > age-dependent URL).

Chi-Square Tests					
	**Value**	**df**	**Asymptotic Significance (2-sided)**	**Exact Sig. (2-sided)**	Exact Sig. (1-sided)
Pearson Chi-Square	10.544[a]	1	0.001		
Continuity Correction[b]	9.024	1	0.003		
Likelihood Ratio	11.055	1	<.001		

Fisher's Exact Test				0.002	0.001
Linear-by-Linear Association	10.395	1	0.001		
N of Valid Cases	71				

a. 0 cells (0.0%) have expected count less than 5. The minimum expected count is 12.68. b. Computed only for a 2x2 table.

Symmetric Measures

		Value	Approximate Significance
Nominal by Nominal	Phi	0.385	0.001
	Cramer's V	0.385	0.001
N of Valid Cases		71	

Mann-Whitney U tests for signficant results described in Table-2

MRC sum score on admission CSF-TP protein > 45 g/L.

Independent-Samples Mann-Whitney U Test Summary

**Total N**	71
Mann-Whitney U	401.500
Wilcoxon W	1941.500
Test Statistic	401.500
Standard Error	72.536
Standardized Test Statistic	-0.531
Asymptotic Sig.(2-sided test)	0.600

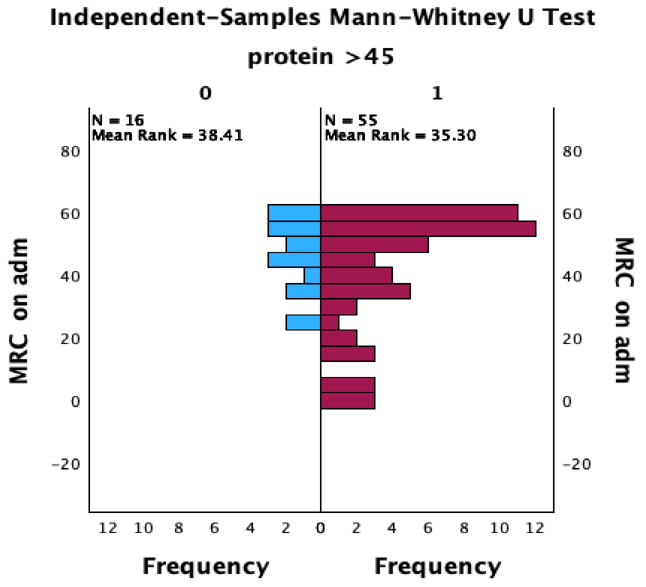

MRC sum score on admission CSF-TP protein > age-dependent URL.

Independent-Samples Mann-Whitney U Test Summary	
**Total N**	71
Mann-Whitney U	475.500
Wilcoxon W	1336.500
Test Statistic	475.500
Standard Error	85.756
Standardized Test Statistic	2.679
Asymptotic Sig.(2-sided test)	<0.001

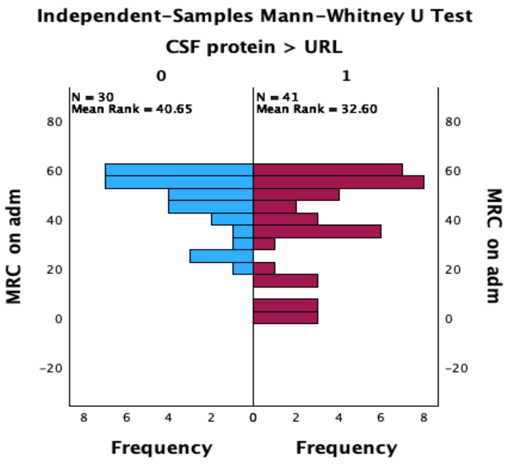

GBS disability score at the time of discharge and CSF-TP protein > 45 g/L.

Independent-Samples Mann-Whitney U Test Summary	
**Total N**	71
Mann-Whitney U	605.500
Wilcoxon W	2145.500
Test Statistic	605.500
Standard Error	71.308
Standardized Test Statistic	2.321
Asymptotic Sig.(2-sided test)	0.026

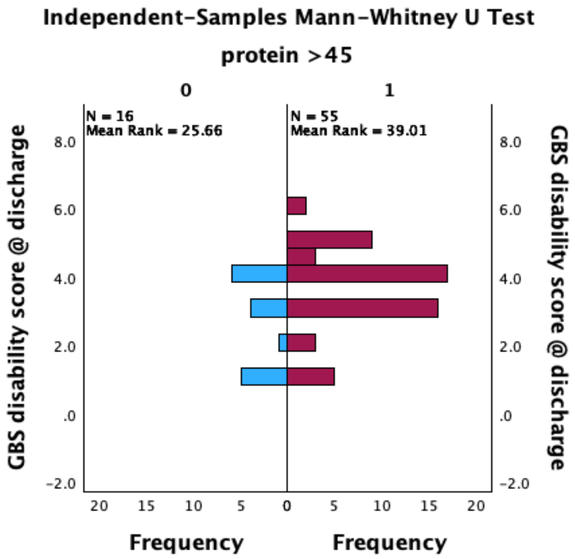

GBS disability score at the time of discharge and CSF-TP protein > age-dependent URL.

Independent-Samples Mann-Whitney U Test Summary	
**Total N**	71
Mann-Whitney U	872.000
Wilcoxon W	1733.000
Test Statistic	872.000
Standard Error	84.305
Standardized Test Statistic	3.048
Asymptotic Sig.(2-sided test)	0.001

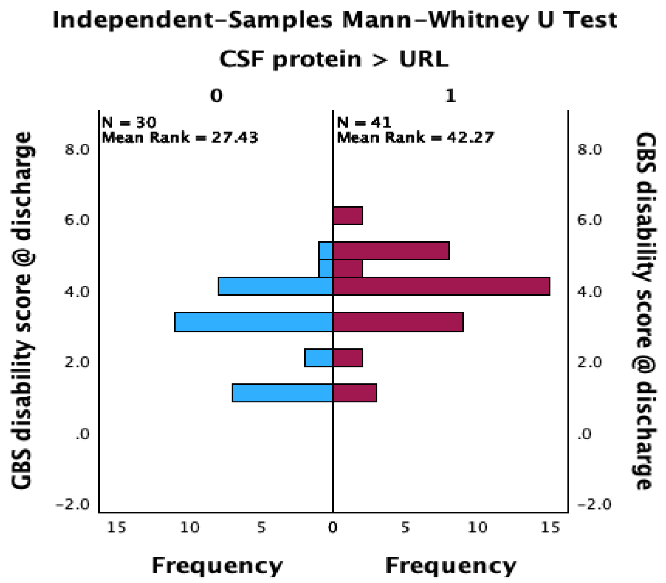

Table S3. Original data of the main results described in Table S3.

1.Linear regression between MRC sum score on admission and independent variables.

ANOVA[a]						
Model		Sum of Squares	df	Mean Square	F	Sig.
1	Regression	4598.849	7	656.978	2.563	0.022[b]
	Residual	16148.616	63	256.327		
	Total	20747.465	70			

a. Dependent Variable: MRC on adm. b Predictors: (Constant), DM, CSF protein, g/L, Age, CSF cell count, Sex M/F, ACD brighton, CSF glucose.

Coefficients[a]								
Model		**Unstandardized Coefficients**		Standardized Coefficients	t	Sig.	95.0% Confidence Interval for B	
		**B**	**Std. Error**	Beta			Lower Bound	Upper Bound
1	(Constant)	50.225	9.632		5.214	<.001	30.977	69.473
	CSF protein, g/L	-0.033	0.012	-0.328	-2.711	**0.009**	-0.058	-0.009
	CSF cell count	0.013	0.043	0.039	0.301	0.765	-0.073	0.099
	CSF glucose	-0.076	0.093	-0.111	-0.821	0.415	-0.262	0.109
	ACD brighton	1.895	5.287	0.048	0.358	0.721	-8.671	12.461
	Age	0.077	0.149	0.065	0.516	0.608	-.221	0.374
	Sex M/F	-1.262	4.137	-0.037	-0.305	0.761	-9.530	7.006
	DM	-11.432	5.583	-0.273	-2.048	0.045	-22.588	-0.275

Dependent Variable: MRC on adm.

5. Linear regression between GBS disability score at discharge and independent variables.

Model		Sum of Squares	df	Mean Square	F	Sig.
**ANOVA**[a]						
1	Regression	28.600	7	4.086	2.500	.025[b]
	Residual	102.949	63	1.634		
	Total	131.549	70			

a. Dependent Variable: GBS disability score @ discharge. b. Predictors: (Constant), DM, CSF protein, g/L, Age, CSF cell count, Sex M/F, ACD brighton, CSF glucose.

Coefficients[a]

Model		Unstandardized Coefficients B	Unstandardized Coefficients Std. Error	Standardized Coefficients Beta	t	Sig.	95.0% Confidence Interval for B Lower Bound	95.0% Confidence Interval for B Upper Bound
1	(Constant)	2.872	0.769		3.734	<.001	1.335	4.409
	CSF protein, g/L	0.002	0.001	0.304	2.503	0.015	0.000	0.004
	CSF cell count	0.005	0.003	0.176	1.340	0.185	-0.002	0.012
	CSF glucose	-0.008	0.007	-0.155	-1.140	0.259	-0.023	0.006
	ACD brighton	0.801	0.422	0.256	1.897	0.062	-0.043	1.644
	Age	0.005	0.012	0.050	0.398	0.692	-0.019	0.028
	Sex M/F	-0.273	0.330	-0.100	-0.826	0.412	-0.933	0.387
	DM	-0.153	0.446	-0.046	-0.344	0.732	-1.044	0.737

Dependent Variable: GBS disability score @ discharge.
